# Predicting Axillary Lymph Node Metastasis of Breast Cancer Using Joint Pre-Trained Fine-Tuning and Contrastive Learning for Contrast-Enhanced Ultrasound

**DOI:** 10.3390/bioengineering12121335

**Published:** 2025-12-08

**Authors:** Rong Huang, Mengshi Tang, Lin Pan, Shaohua Zheng, Shu Chen, Yijie Chen

**Affiliations:** 1 The Academy of Digital China, Fuzhou University, Fuzhou 350001, China; front@fzu.edu.cn; 2 College of Physics and Information Engineering, Fuzhou University, Fuzhou 350001, China; tianmoxi6268@163.com (M.T.); panlin@fzu.edu.cn (L.P.); sunphen@fzu.edu.cn (S.Z.); 3 Clinical Oncology School of Fujian Medical University, Fujian Cancer Hospital, Fuzhou 350014, China

**Keywords:** breast cancer, contrast-enhanced ultrasound, axillary lymph node metastasis assessment, pre-trained fine-tuning, contrastive learning

## Abstract

**Objectives**: Breast cancer is one of the most common malignant tumors among women worldwide, and accurate assessment of axillary lymph node metastasis (ALNM) is crucial for determining treatment strategies. Compared to conventional ultrasound, contrast-enhanced ultrasound (CEUS) can observe blood perfusion and microcirculation changes in primary breast tumors, making it a more ideal diagnostic method for ALNM. **Methods**: To address the issues that CEUS video sequences require a high level of diagnostic experience from clinicians, and the process is time-consuming and labor-intensive, making it challenging to generate large datasets for deep learning models, we proposed a method for predicting breast cancer ALNM that combines pre-trained fine-tuning with contrastive learning. First, within a text-video contrastive learning framework, we fine-tuned pre-trained weights from a large general dataset using a small-scale proprietary dataset. Second, during the fine-tuning phase, we employed random prompt optimization to specifically adjust the text encoder according to the characteristics of breast CEUS videos, and optimized the extracted text and video representations through an adaptive fine-tuning optimizer to better fit the current data distribution. **Results**: Experimental results demonstrated that our method achieved a sensitivity of 0.792 and a specificity of 0.8. **Conclusions**: The study demonstrates that the proposed method effectively leverages CEUS to aid in ALNM diagnosis, highlighting its potential to improve the accuracy of early breast cancer screening and to facilitate the development of more personalized treatment plans for patients.

## 1. Introduction

Breast cancer is a disease that severely threatens women’s health, with the highest incidence and mortality rates among malignant tumors in women [1]. According to the GLOBOCAN 2020 report from the International Agency for Research on Cancer, there were approximately 2.26 million new cases of breast cancer globally each year, accounting for 11.7% of all cancer cases, making it one of the most prevalent types of cancer worldwide [2]. Breast cancer typically first metastasizes and spreads locally, with the axillary lymph nodes often being the first site of cancer cell metastasis [3]. Therefore, the presence of axillary lymph node metastasis (ALNM) is an important indicator in the staging of breast cancer, and its accurate assessment is one of the key factors in determining the treatment options for breast cancer, as well as an important prognostic factor [4].

Conventional ultrasound (US) examination has become a routine clinical method for evaluating breast conditions due to its advantages of being radiation-free, cost-effective, and easy to operate. However, ALNM often manifests as irregular lesion shapes, blurred boundaries, or fissure-like structures on traditional US images. These subtle features are difficult to capture clearly with conventional US, resulting in limited predictive performance for ALNM based on traditional ultrasound [5,6,7,8]. Contrast-enhanced ultrasound (CEUS), as an emerging medical imaging technology, enhances echo signals by using specific contrast agents, significantly improving the observation of blood flow changes and microcirculation information in primary breast tumors, helping doctors better assess the invasiveness and metastatic potential of tumors [9]. Recent studies have further confirmed the added value of CEUS. For instance, Gong et al. [10] demonstrated that integrating dynamic perfusion information from CEUS videos with conventional ultrasound significantly improves the diagnostic accuracy of breast cancer and the prediction of molecular subtypes compared to conventional ultrasound alone. Studies have shown that CEUS has achieved promising results in the diagnosis of various diseases, including thyroid nodules [11], liver lesions [12], and colorectal cancer liver metastases [13]. In the field of breast lesion prediction, Yang et al. [14] combined conventional ultrasound with CEUS video data to develop a time dual-branch network model, which effectively improved the accuracy of breast cancer classification and demonstrated the critical role of temporal information from CEUS videos in the classification task. Chen et al. [15] developed a breast cancer diagnostic model based on CEUS videos that integrated radiologists’ diagnostic experience, showing superior sensitivity and specificity compared to traditional ultrasound-based prediction methods. Additionally, Oshino et al. [16] further confirmed that artificial intelligence techniques can extract key diagnostic features of early breast cancer ALNM from CEUS. Specifically, Kondo et al. [17] demonstrated that machine learning models based on Time-Intensity Curve (TIC) parameters could effectively quantify tumor microcirculation dynamics. However, its application in the diagnosis of ALNM in breast cancer is still relatively limited, mainly due to the following challenges.

(1) In the CEUS images of breast cancer, metastatic and non-metastatic cases exhibit high similarity in certain imaging features, resulting in significant subjectivity and inconsistency when relying on manual visual interpretation. Additionally, the diagnostic process is time-consuming, which affects the efficiency and accuracy of clinical diagnosis [18,19].

(2) Medical imaging data generally faces challenges such as high annotation costs and concerns regarding patient privacy, resulting in a very limited amount of data available for practical applications [20,21]. Therefore, effectively improving the predictive performance of ALNM in breast cancer under the constraint of limited CEUS data is an important challenge currently faced.

To overcome the above-mentioned challenges and to fully leverage the lesion-related information and clinical data contained in the dynamic changes of CEUS video frames, this paper proposed a method for predicting ALNM in breast cancer that combined pre-trained fine-tuning with text-video contrastive learning. This strategy builds upon the fact that the fusion of multimodal information has been demonstrated to effectively enhance the robustness of breast cancer diagnosis. For instance, Rai et al. [22] leveraged the complementary information between mammography and ultrasound imaging to achieve performance superior to single-modality approaches. Similarly, this study aims to integrate the spatiotemporal features of CEUS videos with clinical textual information; by jointly analyzing dynamic visual patterns and semantic textual cues, we seek to achieve more precise prediction. The main contributions are as follows:

(1) Clinical information was incorporated to construct a dual-modal learning framework based on text-video contrastive learning, and the pre-trained weights from a large general dataset are fine-tuned using proprietary small-scale data.

(2) During the fine-tuning stage, a random prompt optimization approach was employed to specifically adjust the text encoder based on the characteristics of breast CEUS videos. Additionally, an adaptive fine-tuning optimizer was utilized to optimize the extracted text and video representations for relevant features, thereby better adapting to the current data distribution.

## 2. Materials and Methods

### 2.1. Materials

Breast contrast-enhanced ultrasound (CEUS) data are inherently difficult to obtain in routine clinical practice. The acquisition process is constrained by the contrast administration procedure, ethical approval and follow-up confirmation, consistency in image interpretation, and the high cost of expert manual annotation, resulting in a scarcity of high-quality clinical CEUS datasets with complete accompanying information. In this study, we focus on a single CEUS dataset to elucidate the role of contrast-enhanced ultrasound imaging in the early prediction of axillary lymph node metastasis in breast cancer, and to verify the feasibility and practical value of this method under conditions of limited real-world clinical data. This study collected ultrasound imaging information from 128 cases diagnosed with malignant breast tumors at Fujian Provincial Cancer Hospital between January 2017 and September 2023, including both conventional ultrasound and contrast-enhanced ultrasound. Additionally, patient clinical information was gathered, which included basic clinical data and parameters from the contrast-enhanced ultrasound. The inclusion criteria for the constructed dataset were as follows: (1) diagnosed with T1 or T2 stage primary breast cancer and treated with breast cancer and axillary lymph node dissection; (2) not having received preoperative treatment. Continuous dynamic images were stored for 2 min. Two doctors with over five years of experience in breast ultrasound diagnosis selected one static key frame image for each lesion. To ensure the consistency and reproducibility of key-frame selection, all cases were independently annotated by two breast ultrasound specialists, each with more than five years of diagnostic experience. When discrepancies arose in determining the key time point, the two readers discussed the case and reached a consensus to finalize the selected frame. Consistency analysis showed good agreement between the two readers. Based on the consensus key frame, the model uniformly sampled 10 input images within a window of ±5 frames. All data were anonymized and used solely for research purposes. The study protocol was reviewed and approved by the Ethics Committee of Fujian Provincial Cancer Hospital (Approval No. K2023-421-01), and written informed consent was obtained from all participants.

### 2.2. Methods

#### 2.2.1. Overall Scheme

This study constructed a dual-branch model designed to jointly analyze and classify CEUS video frames and clinical information texts. The model comprised a video encoding branch and a text encoding branch. In the video encoding branch, we leveraged the ViT (Vision Transformer) [23] architecture and the video processing philosophy of ViViT (Video Vision Transformer) [24] to extract features from CEUS video frames. This architectural design enables the effective mining of critical information in both spatial and temporal dimensions of the video frames, thereby obtaining precise and representative video feature representations. For the text encoding branch, we first subjected the input clinical information texts to randomization and targeted preprocessing operations to enhance the diversity and adaptability of the text data. Subsequently, we employed the Transformer architecture to encode the processed texts. The Transformer architecture, with its powerful parallel processing capabilities and ability to capture long-range dependencies, can efficiently extract deep-level feature representations of clinical information texts, laying a solid foundation for subsequent fusion analysis.

**Figure 1:**
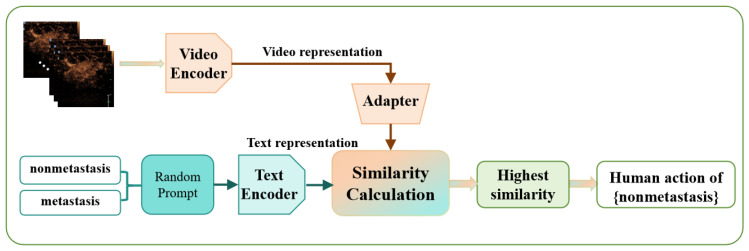
Overall Scheme of the proposed model.

After extracting the video feature representations and text feature representations, we calculate the similarity between them. By comparing the similarity between different text prompts and video features, we select the text prompt with the highest similarity as the final predicted category output, thereby achieving accurate classification of CEUS videos and the associated interpretation of clinical information. Moreover, in the challenging scenario of few-shot learning, to significantly enhance the model’s predictive performance, we introduce a pre-trained adaptive fine-tuning optimizer in both the video encoding branch and the text encoding branch. This optimizer can fully leverage the advantages of pre-trained models under limited sample conditions and, through adaptive fine-tuning, enable the model to better adapt to the specific task and data distribution at hand, thereby further optimizing the model’s performance. As shown in Figure 1, this is the overall architecture diagram of the innovative method proposed in this study, clearly illustrating the collaborative working relationship between various modules and the flow and processing of data.

#### 2.2.2. Data Preprocessing

**Figure 2:**
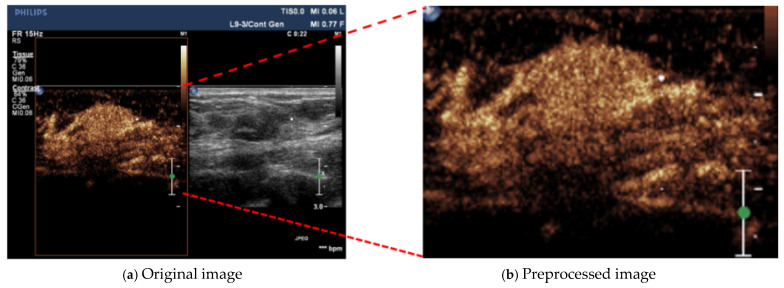
Data Preprocessing illustration.

In the original ultrasound images, there are additional information areas, such as device information and parameter settings, as shown in Figure 2. If the raw images are used directly for training, they may interfere with the network and affect prediction performance. Therefore, during data preprocessing, it is necessary to remove these additional information areas as well as the surrounding black background regions. The preprocessing steps are as follows: (1) Apply a threshold of 50 to create a binary image; (2) Use the connected component segmentation method to select the bounding rectangle with the largest connected component as a mask to crop the original image, obtaining the optimal central region.

#### 2.2.3. Adaptive Fine-Tuning Optimizer

**Figure 3:**
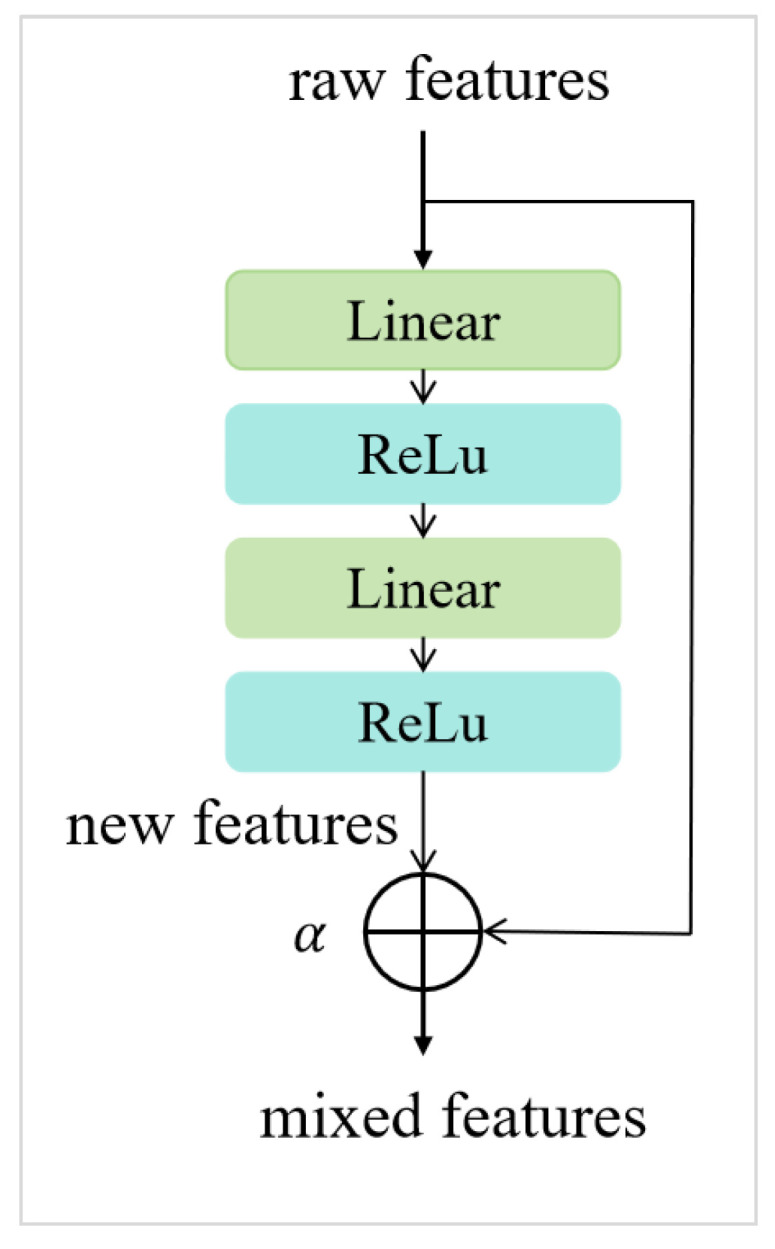
The structure of the adaptive fine-tuning optimizer.

The pre-training and fine-tuning strategy is widely applied in various downstream tasks. However, due to the large number of parameters in pre-trained models, there is a tendency to overfit in scenarios with limited samples. This study implemented an Adaptive Fine-Tuning Optimizer (AFTO) [25] to address this issue. The core idea was to introduce lightweight feature adapters to achieve efficient fine-tuning of the pre-trained model. The structure of the fine-tuning optimizer used in this study and its application are illustrated in Figure 3. The general features obtained from pre-training undergo two linear transformations and a nonlinear activation function (ReLU) to produce new feature representations. These new features are then mixed with the original features through residual connections to obtain optimized features. Through this operation, the network can retain the rich knowledge accumulated during the pre-training phase while further learning feature representations that are more relevant to the task of predicting axillary lymph node metastasis in breast cancer. This strategy not only effectively avoids the overfitting problem caused by limited fine-tuning data but also significantly enhances the model’s generalization ability for few-shot fine-tuning learning in specific scenarios.

During feature mixing, the mixing factor α is used to control the blending ratio of the original features and the new features, as shown in Equation (1). Here, $F_{mix}$ represents the optimized features after mixing, $F_{new}$ is the new features obtained after the Adapter, and $F_{raw}$ is the original features.

*F_mix_* = α·*F_new_* + (1 − α)·*F_raw_* (1)

To address the limitation of manual setting of the mixing coefficients of fine-tuning optimizers in the joint scenario of multimodal encoders, which requires a large number of experiments to determine the optimal parameter ratios, we adopted dynamic mixing coefficient optimization. We parameterized the mixing coefficients and embedded them into the network structure, allowing them to be automatically adjusted through backpropagation during training. This approach not only maintained the flexibility of the model but also significantly reduced experimental costs, providing a more efficient and practical method for fine-tuning optimization strategies.

#### 2.2.4. Text Random Prompt Optimization

In the CLIP (Contrastive Language-Image Pre-training) model [26], text descriptions are typically designed based on the scenes corresponding to the pre-training data. To better adapt to the current task when transferring, it is necessary to construct corresponding descriptive texts according to the classification labels of the target task, such as “A photo of {label}”, and then input these texts into the text encoder to obtain the corresponding text features. The breast cancer ALNM prediction task is a specific direction in the field of medical image processing, using CEUS video data. There is a significant difference compared to the pre-trained weights obtained from conventional data. During fine-tuning, this difference may have a certain impact on the results. Therefore, the text description needs to be modified specifically. To this end, random prompts were introduced for optimization to enhance the model’s robustness and reduce the risk of overfitting.

Specifically, when setting the prompt, we constructed a text prompt pool, which consisted of three types of prompts: pre-prompt, mid-prompt, and post-prompt. Each type of prompt contained multiple descriptions of the video data, and these descriptions included content tailored to the breast cancer ALNM prediction task, instead of using only generic text content. As shown in Table 1, the category label is filled in the “{}”. Label types include: Basic clinical data, Histopathological biomarkers, Contrast-enhanced ultrasound parameters, Conventional ultrasound parameters, and model labels for classification. Due to space limitations, the detailed prompts and label categories are provided in the Supplementary Materials.

During training, the network first randomly selected a prompt for each data instance from the predefined text prompt pool, and then inputted it into the text encoder for text feature extraction. Through this text prompt pool and random selection strategy, the text encoding module can learn more diverse and more targeted text features, thereby enhancing the model’s adaptability and generalization ability for the specific task of breast cancer axillary lymph node metastasis prediction.

#### 2.2.5. Text Encoder

**Figure 4:**
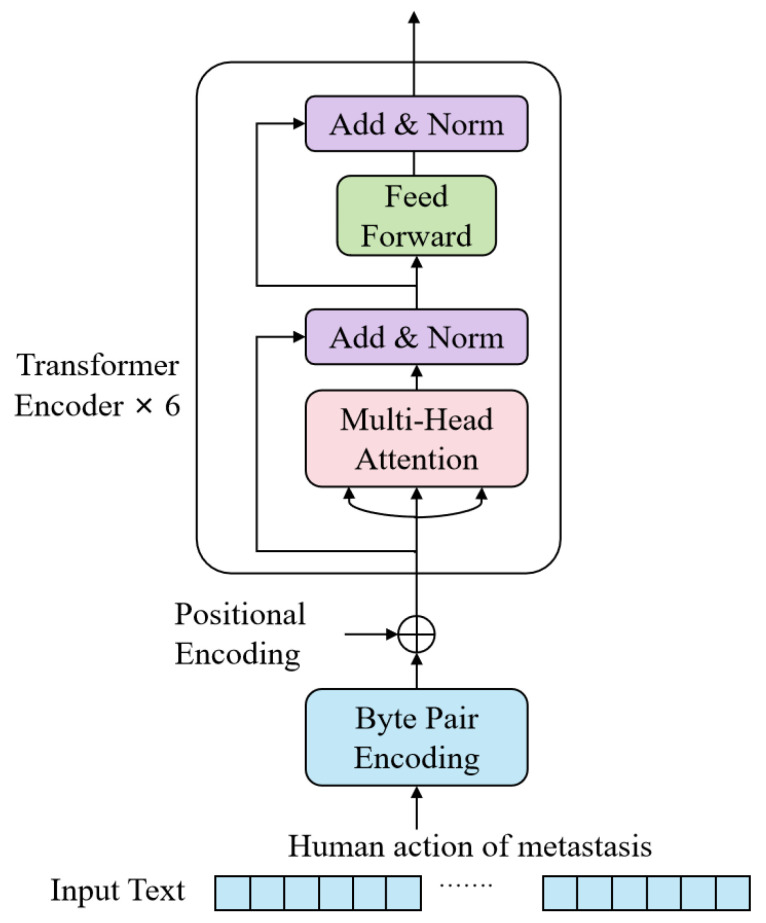
The structure of the Text Encoder.

The text encoder was based on the encoder module of the Transformer. Its core idea is to convert the input natural language text into low-dimensional vector representations so that it can be aligned with the output of another branch, the video encoder, for subsequent similarity computation. The structure of the text encoder, as shown in Figure 4, consists of six Transformer encoder layers. Each layer is composed of two main modules: Multi-Head Attention and Feed-Forward Neural Network.

Text encoding included both text encoding and positional encoding. The text encoding used Byte Pair Encoding (BPE) to segment the text into a series of fixed-size subword units, which were mapped to a predefined vocabulary. Then, each input subword unit was embedded into a fixed-dimensional vector space, where the dimension matches the size of the model’s hidden layers. Subsequently, these embedding vectors were enhanced by positional encoding (PE) to retain the positional information within the text sequence. The entire text encoder consisted of 6 such Transformer layers, outputting a fixed-dimensional vector, which was then linearly projected into a multimodal embedding space for contrastive learning with the output of the image encoder.

#### 2.2.6. CEUS Video Encoder

**Figure 5:**
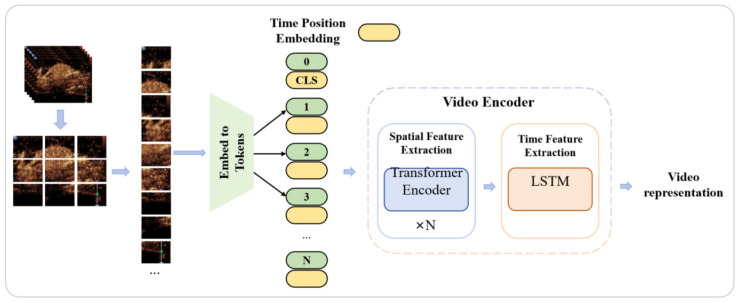
The structure of the CEUS Video Encoder.

In order to leverage the pre-trained weights from the CLIP model, this paper modifies the Vision Transformer (ViT) architecture employed in CLIP to enable the processing of video frame data. Specifically, the modified architecture extracts video semantic features that encapsulate both spatial and temporal information. This is achieved by incorporating the video frame processing approach from the Video Vision Transformer (ViViT) and the positional embedding method used in the text encoder to maintain the sequential order of video frames [24]. The overall structure of the proposed architecture is illustrated in Figure 5.

First, video frames were uniformly sampled, and each frame was processed using the ViT approach, where it was divided into fixed-size patches, and each patch was converted into a token. Then, a corresponding temporal positional embedding was added to each token to indicate its temporal position within the video. Finally, all the tokens from the frames were concatenated together. In this way, each token contained not only spatial information but also temporal information, as shown in Equations (2)–(4).

Tokens = [frame_1_, frame_2_, …, frame_i_] (2)

frame_i_ = [token_i,1_, token_i,2_, …, token_i,j_] (3)

token_i,j_ = token_i,j_’ + TPE (4)

Here, Tokens refers to the final sequence of tokens obtained, frame_i_ represents the sequence of all tokens contained in a single video frame, token_i*,*j_ denotes an individual token within a single video frame, and TPE stands for Temporal Position Embedding.

Next, the token sequence will be fed into a video encoder composed of a spatial feature extraction module based on ViT and a temporal feature extraction module based on a Long Short-Term Memory (LSTM) model. The spatial feature extraction module, which is based on the ViT architecture, fully utilizes the pretrained weights obtained from CLIP and consists of six layers of Transformer Encoders. The structure of each Transformer Encoder layer is similar to that of the Transformer architecture in the text encoder. Through this series of operations, the network can effectively capture long-range dependencies within the images, thereby generating more powerful spatial feature representations. During the encoding process, the temporal position embeddings added to each patch token enable the model to perceive temporal information and, by leveraging the multi-head self-attention mechanism of the Transformer, implicitly learn the temporal features between frames.

The temporal features learned solely through the multi-head attention mechanism in Transformer are insufficient for video classification tasks. Behaviors in videos typically involve complex dynamic changes across multiple frames, which require more robust temporal feature representations to capture. Therefore, it is necessary to introduce a dedicated temporal feature extraction module to explicitly extract more powerful temporal features between video frames, thereby enhancing the performance of the model. To this end, the LSTM model was introduced as the temporal feature extractor. Specifically, the spatial feature representations that already contained temporal positional information of video frames were fed into the LSTM model in their temporal order. In this way, the LSTM model was able to progressively capture the temporal relationships between frames, thereby strengthening the temporal feature representation and significantly improving the performance of video classification.

#### 2.2.7. Loss Functions

The loss function used the KL divergence loss to compute the difference between the similarity distributions of video-to-text (V → T, p^x2y^) and text-to-video (T → V, p^y2x^) and the true distributions (q^x2y^, q^x2y^), as shown in Equation (5).

L = 1/2 E_(x,y)~D_ [KL(p^x2y^(x), q^x2y^(x) + KL(p^y2x^(y), q^y2x^(y)] (5)

## 3. Results

### 3.1. Experimental Environment and Parameter Settings

All experiments were conducted on an NVIDIA Tesla P100 device with 16 GB of GPU memory, using the PyTorch 2.0.1 training environment. The training hyperparameter configuration is detailed in the Supplementary Materials. To prevent data leakage, the five-fold splits were performed at the patient level with class-balanced stratification, ensuring that all samples from the same patient were confined to one fold and that the ratio of metastatic to non-metastatic cases remained consistent across all folds. During the training process, five-fold cross-validation was employed, along with data augmentation and early stopping strategies to mitigate overfitting. The model was trained for a minimum of 50 epochs, with a batch size of 4, and the optimizer used was AdamW.

### 3.2. Experimental Results and Analysis

To verify the effectiveness of the proposed method, we conducted comparative experiments with five mainstream methods currently in use. These methods are: (1) Convolutional Neural Network (CNN)-based video classification methods, including R(2+1)D [27], DenseNet3D [28]; (2) Transformer-based video classification method, namely Uniformer [29]; and (3) Contrastive learning-based video classification method, namely CPCTR [30]. The results of the comparative experiments are shown in Table 2.

Among the compared methods, the CNN-based DenseNet3D, ResNet3D, and R(2+1)D exhibited similar performance and outperformed the Transformer-based Uniformer. This indicates that these classical 3D convolutional network architectures can provide relatively stable performance. However, when dealing with the more challenging CEUS video data, their potential for performance improvement is relatively limited. In contrast, Uniformer performed relatively weakly in these modalities, likely due to the Transformer framework’s dependence on large-scale datasets during training. Consequently, its performance is constrained when the available data volume is limited. Despite this, the proposed method in this paper, based on the Transformer framework, effectively mitigated the impact of limited data on model performance by introducing large-scale pre-trained weights. This demonstrates the effectiveness of the pre-training and fine-tuning strategy. Additionally, CPCTR outperformed the aforementioned CNN-based and Transformer-based methods, further validating the effectiveness of the contrastive learning strategy in enhancing model performance.

**Figure 6:**
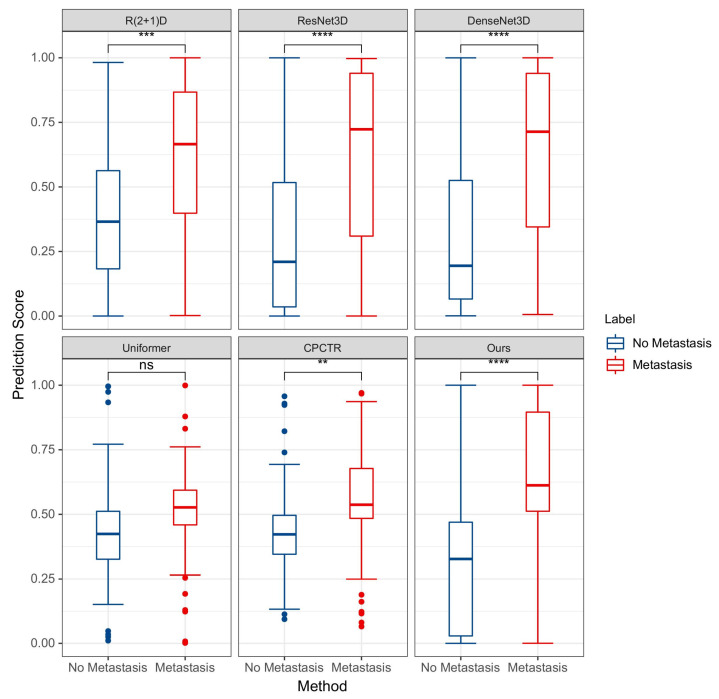
Box plot of prediction results for CEUS data. (ns: *p* > 0.05; **: *p* ≤ 0.01; ***: *p* ≤ 0.001; ****: *p* ≤ 0.0001).

Figure 6 presents the box plot of the prediction results for axillary lymph node metastasis of breast cancer using the CEUS modality, showcasing the distribution of prediction scores from various methods and indicating the corresponding statistical significance differences. The results demonstrate that the proposed method shows significant statistical significance (*p* < 0.0001) when predicting based on CEUS data, and there is no overlap between the boxes of metastatic and non-metastatic samples, confirming that this method has a more pronounced discriminative ability compared to others. Next is CPCTR, which has a relatively concentrated distribution of prediction scores, but there is slight overlap between the boxes, and the surrounding discrete points indicate the possibility of outliers in certain cases. Although the distribution of Uniformer is similar to that of CPCTR, the *p* > 0.05 indicates that its results lack statistical significance. R(2+1)D, ResNet3D, and DenseNet3D show a high degree of overlap in prediction scores, suggesting that these three methods have a weaker ability to differentiate between metastatic and non-metastatic samples.

**Figure 7:**
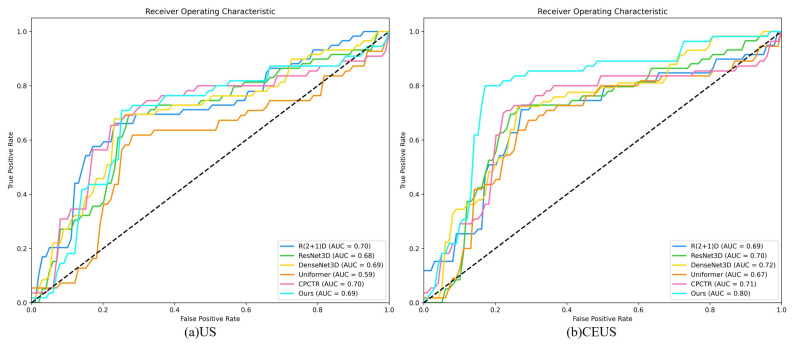
ROC curve plots of the prediction results. The dashed diagonal line represents the random guess baseline, where the true positive rate equals the false positive rate, corresponding to AUC = 0.5.

Figure 7 presents the ROC curves and AUC values of various methods for predicting axillary lymph node metastasis of breast cancer using the CEUS modality. In comparison, the proposed method demonstrates the best performance in the CEUS modality, achieving an AUC of 0.80. The R(2+1)D, ResNet3D, DenseNet3D, and CPCTR methods perform moderately, while Uniformer shows the poorest performance.

**Figure 8:**
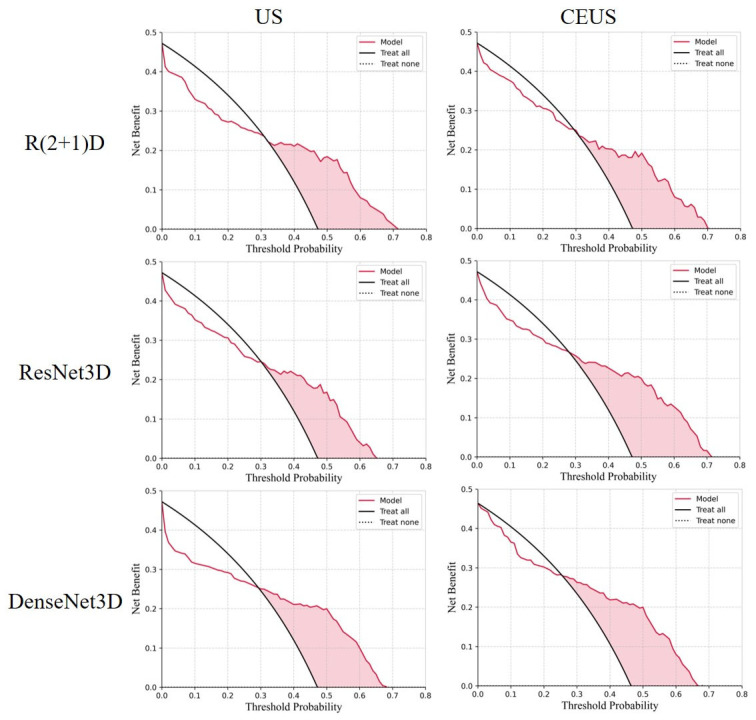

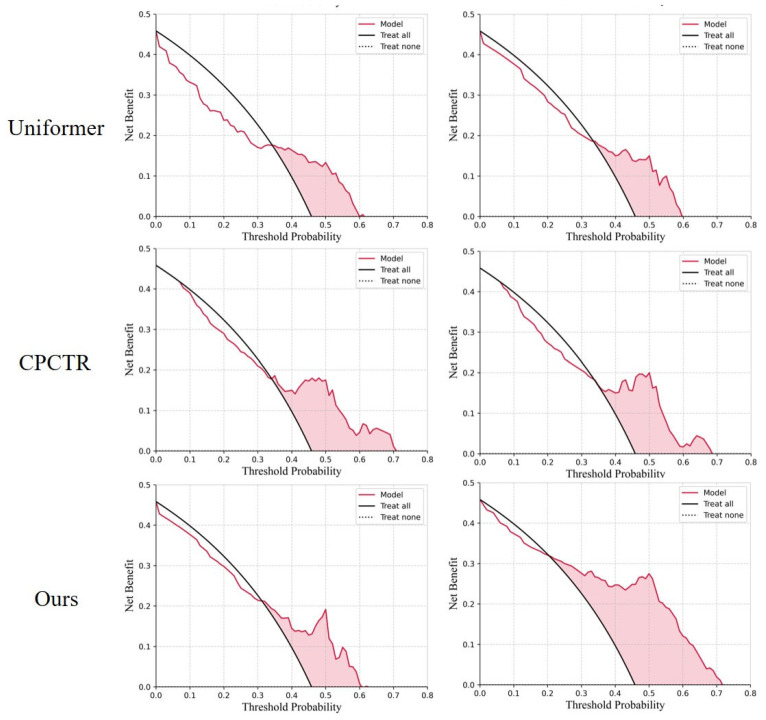
Decision curve analysis of the prediction results. “Treat none” is a baseline representing the scenario where no intervention is performed on any samples. Its core characteristic is a constant net benefit of 0, thus forming a horizontal line in the x-axis.

Figure 8 presents the decision analysis curves of various methods for predicting ALNM in breast cancer using CEUS modality data. The proposed method significantly outperforms other methods in terms of performance. Within the threshold probability interval [0.21, 0.71], the proposed method consistently achieves positive net benefits, with the widest range of positive net benefits and the highest maximum net benefit. This indicates that the proposed method’s prediction results are highly stable and practical across different decision thresholds. In contrast, the three CNN-based 3D convolutional methods (DenseNet3D, ResNet3D, and R(2+1)D) perform next best. Although Uniformer and CPCTR are comparable to the CNN-based methods in terms of the range of positive net benefits and maximum net benefits, their model decision curves (Model) exhibit significant fluctuations. Particularly, Uniformer shows notable fluctuations in the interval [0.5, 0.55], and CPCTR in the interval [0.55, 0.6]. This suggests that the decision stability of these two methods is relatively poor in certain threshold intervals, and their applicability may be somewhat limited. This phenomenon may be attributed to the instability of complex models when predicting with limited and relatively complex datasets. Despite their theoretically stronger fitting capabilities, complex model structures are prone to overfitting or significant fluctuations when data is limited, thereby affecting their reliability in practical decision-making.

### 3.3. Experimental Result of the Module Ablation

To verify the effectiveness of different modules, the combinations in the ablation experiments include:

(1) Baseline: Using only CLIP with contrast-enhanced ultrasound video frames as input;

(2) Baseline + RP: Adding Random Prompt (RP) optimization to the Baseline to assess the impact of textual information on model performance;

(3) Baseline + AFTO (video): Incorporating the Adaptive Fine-Tuning Optimizer (AFTO) in the video encoder based on the Baseline;

(4) Baseline + AFTO (text): Incorporating the Adaptive Fine-Tuning Optimizer (AFTO) in the text encoder based on the Baseline;

(5) Baseline + AFTO (both): Adding the Adaptive Fine-Tuning Optimizer (AFTO) to both branches based on the Baseline;

(6) Baseline + RP + AFTO: Combining both RP and AFTO in the Baseline, which represents the comprehensive method proposed in this chapter, to fully validate its overall performance.

**Figure 9:**
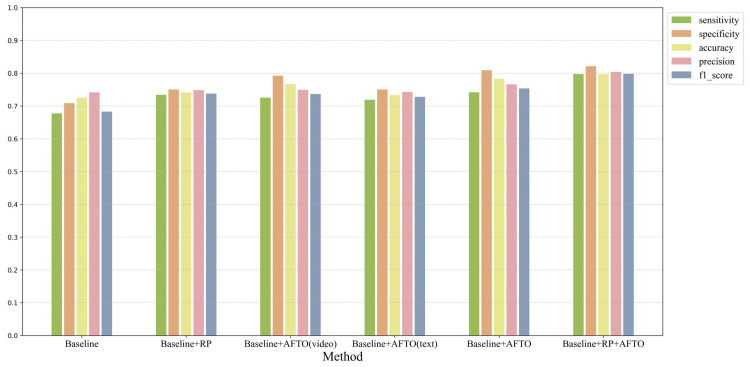
The result of Module ablation.

The results of the module ablation experiments are shown in Figure 9. Compared with the Baseline, the model’s various performance metrics improved after introducing Random Prompt optimization, although the improvement was relatively small. However, this result fully demonstrates the positive impact of diverse textual input on the model. When AFTO was added only to the video encoder, the model’s metrics showed significant improvement compared to the Baseline, especially in specificity, which increased from 0.7087 to 0.7927. This indicates that AFTO’s optimization of video features can effectively enhance the model’s performance, highlighting the critical role of video feature optimization. In contrast, when AFTO was added only to the text encoder branch, the model’s performance improvement was not significant. This suggests that, in this task, optimizing video features is more important than optimizing text features. Furthermore, when AFTO was introduced to both the video and text branches simultaneously, the model’s overall performance was further enhanced. This indicates that joint optimization of both branches can complement the features of the two modalities, thereby more fully leveraging the targeted fine-tuning capabilities of AFTO.

### 3.4. Experimental Result of the Video Frame Reduction Ablation

**Figure 10:**
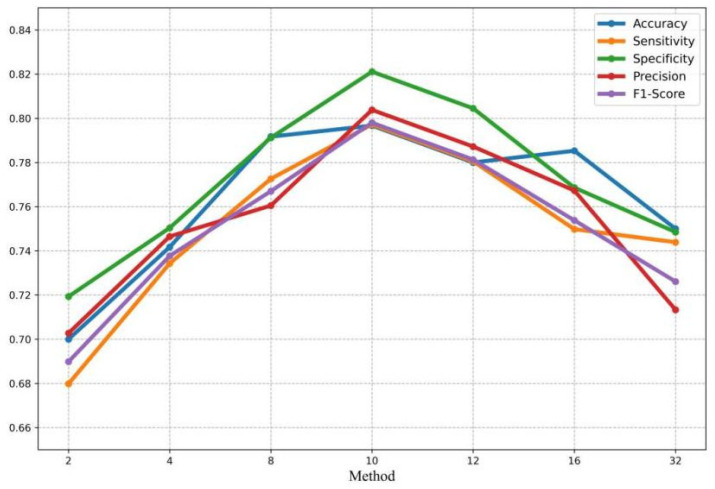
The ablation results of video frames.

Since the proposed model takes a certain number of video frames as input, we conducted ablation experiments on the number of video frames to assess its impact on model performance and determine the optimal number of frames. The experiments were tested with 2, 4, 8, 10, 12, 16, and 32 frames, respectively, and the results are shown in Figure 10. Initially, the model performance gradually improved with the increase in the number of input frames, reaching the best effect when 10 video frames were selected. When the number of frames continued to increase, the overall performance of the model showed a downward trend.

**Figure 11:**
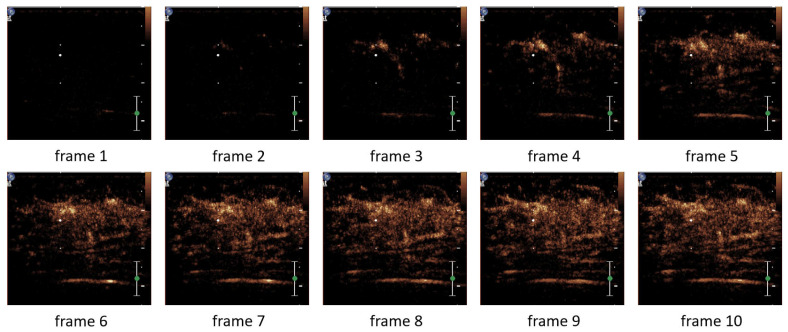
10-frame video example.

**Figure 12:**
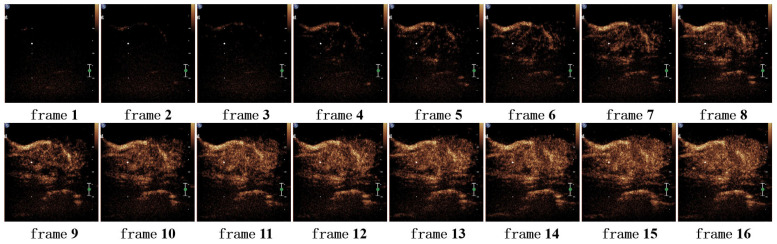
16-frame video example.

The optimal model performance at 10 frames may be attributed to the fact that a higher number of frames can lead to the loss of some effective information. Figure 11 and Figure 12 show the comparison of video frames for the same sample when 10 frames and 16 frames are sampled, respectively. When 10 frames are sampled, there are significant changes in the lesion morphology between each frame and the previous one (as indicated by the red arrows in the figures), and these changes can provide effective temporal information to the network. However, when the number of frames reaches 16, it is observed that there is no significant change in the lesion morphology in the last few frames, and the excessive number of frames can instead impose a burden on the model.

## 4. Discussion

Experimental results demonstrate that when predicting ALNM of breast cancer based on CEUS video data, the proposed method in this study achieves a specificity, precision, and F1-score all exceeding 0.8, with the sensitivity and accuracy reaching 0.7972 and 0.7967, respectively. All evaluation metrics exhibit excellent balance. These results confirm that the proposed method outperforms existing comparative approaches, offering a potentially effective tool for the accurate assessment of breast cancer ALNM in clinical practice.

Ablation experiments further verified that the combination of the random prompt optimization strategy and the AFTO-oriented targeted fine-tuning for both video and text branches enables the model to achieve optimal performance across all evaluation metrics. This result indicates that the diverse text inputs generated by random prompt optimization complement the branch-specific feature optimization driven by AFTO: the former effectively enriches the dimensionality and generalization ability of text representations learned by the model, while the latter enhances the feature discriminability and matching degree of video and text branches for the target task (breast cancer ALNM prediction) through targeted fine-tuning. The synergistic effect of these two components ultimately achieves a systematic improvement in the comprehensive prediction performance of the model.

Despite promising results, several limitations should be acknowledged in this study. First, our proposed method is primarily intended for breast cancer screening as an auxiliary diagnostic tool and has not yet reached the stage of clinical translation or direct application. Further validation in prospective clinical trials is necessary to confirm its efficacy and robustness in diverse patient populations. Second, the current dataset, although comprehensive, may have limitations in terms of sample size and population diversity, which could affect the generalizability of the model to broader clinical settings. Third, while the model leverages advanced contrast-enhanced ultrasound (CEUS) imaging, variability in imaging protocols and operator dependency could influence performance in real-world clinical environments. Finally, integration with existing clinical workflows and evaluation of cost-effectiveness remain to be explored before routine clinical adoption can be considered.

Future work will focus on addressing these limitations by expanding datasets, conducting multicenter prospective studies, and collaborating closely with clinicians to facilitate clinical translation.

## 5. Conclusions

The proposed method for predicting breast cancer ALNM that integrates pre-trained fine-tuning with contrastive learning demonstrates significant potential for improving diagnostic accuracy. By leveraging a text-video contrastive learning framework and employing random prompt optimization tailored to breast CEUS videos, our approach effectively addressed the challenges associated with the complex and time-consuming nature of CEUS data analysis. The experimental results highlight the effectiveness of this method, achieving a sensitivity and specificity of 0.8. This indicates that our approach can provide objective and reliable diagnostic support, potentially aiding clinicians in making more informed treatment decisions for breast cancer patients. Future work may focus on further optimizing the model and expanding the dataset to enhance its generalizability and clinical applicability.

## Figures and Tables

**Table 1:** Text prompt word example.

Pre-Prompt	Mid-Prompt	Post-Prompt
{}, an action	video classification of {}, a disease	a video of action {}
{} this is an disease	the disease is {}, look here	a sample of disease {}
{}, a video of symptom	Can you recognize {}? a Symptom of disease	Human of {}
……	……	……

**Table 2:** Experimental results of different methods.

Data	Method	Sen	Spe	Acc	Pre	F1
CEUS	R(2+1)D [27]	0.7124 ± 0.1258	0.7367 ± 0.0977	0.7200 ± 0.0248	0.6966 ± 0.1194	0.6924 ± 0.0790
	DenseNet3D [28]	0.7203 ± 0.1134	0.7477 ± 0.1064	0.7360 ± 0.0727	0.7061 ± 0.1453	0.7034 ± 0.1138
	Uniformer [29]	0.6691 ± 0.1346	0.6875 ± 0.1741	0.6917 ± 0.1222	0.6660 ± 0.0738	0.6625 ± 0.1032
	CPCTR [30]	0.7038 ± 0.1200	0.7733 ± 0.0957	0.7520 ± 0.0800	0.7235 ± 0.1491	0.7121 ± 0.1302
	Ours	**0.7972 ± 0.0927**	**0.8211 ± 0.0629**	**0.7967 ± 0.0439**	**0.8038 ± 0.0582**	**0.7980 ± 0.0873**

The best results are indicated in bold and can be retained.

## Data Availability

The datasets are not publicly available due to restrictions of the hospital.

## Supplementary Materials

The following supporting information can be downloaded at: https://www.mdpi.com/article/10.3390/bioengineering12121335/s1, Supplementary File S1: Category of Labels & Super Parameter; Supplementary File S2: Confusion Matrix of Inference; Supplementary File S3: Comparison between actual cases and predictions for axillary lymph node metastasis in T1–T2 breast cancer; Supplementary File S4: Computational cost of training and inference.
