# Supplementary material for: Predicting Axillary Lymph Node Metastasis of Breast Cancer Using Joint Pre-Trained Fine-Tuning and Contrastive Learning for Contrast-Enhanced Ultrasound
Source: Bioengineering (Basel). 2025 Dec 8;12(12):1335. doi: 10.3390/bioengineering12121335 (PMC12729765; doi:10.3390/bioengineering12121335)
Supplement: Supplementary file 1 [file bioengineering-12-01335-s001.zip › Supplementary S1. Category of Labels & Super Parameter .pdf]

Supplement S1.

Table S1. Category of Labels

Category	Variable	Text prompt value
Basic clinical data	name	patient name
	age	age (years)
	distance to nipple	distance to nipple (mm)
	distance to skin	distance to skin (mm)
	distance to pectoralis major	distance to pectoralis major (mm)
	location 1	0 = upper-outer quadrant, 1 = others
	location 2	0 = upper-outer, 1 = lower-outer, 2 = lower-inner, 3 = upper-inner, 4 = retro-areolar
	maximum diameter	tumor maximum diameter (mm)
	ILI	image label ID
	T1_T2	T1 / T2 stage
	type	0 = in-situ, 1 = invasive
	lymphovascular invasion	0 = absent, 1 = present
	grade	1 = grade I, 2 = grade II, 3 = grade III, 4 = undetermined, 5 = DCIS, 6 = others
Histopathological biomarkers	ER	0 = negative, 1 = positive, 2 = not available
	PR	0 = negative, 1 = positive, 2 = not available
	HER2	0 = negative, 1 = positive, 2 = not available
	Ki67	0 = < 14 %, 1 = ≥ 14 %, 2 = not available
	molecular subtype	0 = luminal A, 1 = luminal B, 2 = triple-negative, 3 = HER2-enriched, 4 = not available
Contrast-enhanced ultrasound parameters	enhancement intensity	0 = iso-/hypo-, 1 = hyper-
	enhancement phase	0 = slow-/iso-, 1 = fast wash-in
	post-enhancement size change	0 = unchanged, 1 = enlarged, 2 = indistinct
	enhancement pattern	0 = non-centripetal, 1 = centripetal
	enhancement homogeneity	0 = heterogeneous, 1 = homogeneous
	enhancement completeness	0 = incomplete, 1 = complete
	crab-leg sign	0 = absent, 1 = present
	feeding vessel	0 = absent, 1 = present
	post-enhancement margin	0 = clear, 1 = unclear, 2 = indistinct
	post-enhancement shape	0 = regular, 1 = irregular, 2 = indistinct
Conventional ultrasound parameters	BI-RADS category	BI-RADS final category
	angular margin	0 = absent, 1 = present
	lobulated contour	0 = absent, 1 = present
	spiculation	0 = absent, 1 = present
	hyperechoic halo	0 = absent, 1 = present
	echo pattern	0 = hypoechoic, 1 = isoechoic, 2 = hyperechoic
	micro-calcification	0 = absent, 1 = present
Model label	label	0 = no axillary lymph-node metastasis, 1 = metastasis present

Table S2. Super Parameter of Clips

Category	Parameter	Value
**Backbone**	Architecture	CLIP ViT-B/32
	Patch size	32
	Input resolution	512 × 512
	Pre-trained weights	OpenAI CLIP (frozen)
**Adapters**	Adapter type	text / image / both
	Text adapter strength	0.5
	Image adapter strength	0.5
	Joint adapter strength	0.5
**Video Sampling**	Frames per clip (T)	8
	Sampling method	Uniform sampling
	Temporal augmentation	Random shift (train only)
**Batching**	Batch size	1
	Number of GPUs	1 (NVIDIA P100, 8GB)
	Precision	Mixed FP16
**Data Augmentation (Train)**	Spatial crop	Random resized crop to 512 × 512
	Flip	Horizontal flip, p = 0.5
	Color jitter	Disabled
	RandAugment	N = 2, M = 9
	Normalization	CLIP mean/std
**Data Augmentation (Val)**	Resize	Center crop to 512 × 512
	Flip	Disabled
	Normalization	CLIP mean/std
**Loss Function**	Type	Symmetric KL-divergence loss
	Loss formula	(KL(img→txt) + KL(txt→img)) / 2
**Optimizer**	Optimizer	AdamW
	Learning rate	1.00E-05
	Weight decay	0.01
	Adam betas	(0.9, 0.98)
**LR Scheduler**	Schedule	Cosine annealing
	Warm-up	5 epochs
	Total epochs	30
**Inference**	Text prompts	Multiple templates averaged
	Similarity	L2-normalized cosine similarity
	Output	Softmax over class scores
