# Supplementary material for: Predicting Axillary Lymph Node Metastasis of Breast Cancer Using Joint Pre-Trained Fine-Tuning and Contrastive Learning for Contrast-Enhanced Ultrasound
Source: Bioengineering (Basel). 2025 Dec 8;12(12):1335. doi: 10.3390/bioengineering12121335 (PMC12729765; doi:10.3390/bioengineering12121335)
Supplement: Supplementary file 1 [file bioengineering-12-01335-s001.zip › Supplementary S2. Confusion Matrix of Inference .pdf]

Supplement S2.

Table S3. Confusion matrix of inference results

The inference was performed on a set of 120 historical cases, independently reviewed by expert clinicians, which were distinct from the training and test sets. Among these cases, 55 were positive (45.8%) and 65 were negative (54.2%).

	**Predicted:Metastasis**	**Predicted:Non-metastasis**	**Actual Total**
**Actual:Metastasis**	44	11	55
**Actual:Non-metastasis**	11	54	66
**Predicted Total**	55	65	120

Table S4. Performance metrics of the proposed network on the external validation set

Metric	Formula	Value (%)
Sensitivity (Recall^{+})	TP / (TP + FN)	80.00
Specificity (Recall^{-})	TN / (TN + FP)	83.08
Precision (PPV)	TP / (TP + FP)	80.00
Negative Predictive Value (NPV)	TN / (TN + FN)	83.08
Overall Accuracy	(TP + TN) / (TP + TN + FP + FN)	81.67

The overall classification accuracy was 81.67%.That proposed our network can still deliver effective and reliable inference results when trained on a small amount of data.
