# Supplementary material for: Predicting Axillary Lymph Node Metastasis of Breast Cancer Using Joint Pre-Trained Fine-Tuning and Contrastive Learning for Contrast-Enhanced Ultrasound
Source: Bioengineering (Basel). 2025 Dec 8;12(12):1335. doi: 10.3390/bioengineering12121335 (PMC12729765; doi:10.3390/bioengineering12121335)
Supplement: Supplementary file 1 [file bioengineering-12-01335-s001.zip › Supplementary S4 .Computational cost of training and inference .pdf]

Supplement S4.

**Table S5.** Computational cost of training and inference

| Model | Params (M) | TM (MB) | TT (s) | IM (MB) | IT (s) |
|---|---|---|---|---|---|
| Ours | 142 | 9106 | 286 | 1536 | 29 |

Evaluation of Model Efficiency, Include Model Parameters (Params), Training Memory (Tm), Training Time (Tt), Inference Memory (Im) And Inference Time (It).
